# Social and Behavioral Determinants of Perceived Insufficient Sleep

**DOI:** 10.3389/fneur.2015.00112

**Published:** 2015-06-05

**Authors:** Michael A. Grandner, Nicholas J. Jackson, Bilgay Izci-Balserak, Rebecca A. Gallagher, Renee Murray-Bachmann, Natasha J. Williams, Nirav P. Patel, Girardin Jean-Louis

**Affiliations:** ^1^Center for Sleep and Circadian Neurobiology, Perelman School of Medicine, University of Pennsylvania, Philadelphia, PA, USA; ^2^Behavioral Sleep Medicine Program, Department of Psychiatry, Perelman School of Medicine, University of Pennsylvania, Philadelphia, PA, USA; ^3^Quantitative Psychology Program, Department of Psychology, University of Southern California, Columbia, SC, USA; ^4^Division of Sleep Medicine, Perelman School of Medicine, University of Pennsylvania, Philadelphia, PA, USA; ^5^School of Nursing, University of Pennsylvania, Philadelphia, PA, USA; ^6^Department of Medicine, State University of New York Downstate Medical Center, Brooklyn, NY, USA; ^7^Division of Internal Medicine, Center for Healthful Behavior Change, New York University Medical Center, New York, NY, USA; ^8^Department of Medicine, Reading Hospital and Medical Center, Reading, PA, USA

**Keywords:** insufficient sleep, behavioral, social determinants, sleep duration, cardiometabolic disease, poor health

## Abstract

Insufficient sleep is associated with cardiometabolic disease and poor health. However, few studies have assessed its determinants in a nationally representative sample. Data from the 2009 behavioral risk factor surveillance system were used (*N* = 323,047 adults). Insufficient sleep was assessed as insufficient rest/sleep over 30 days. This was evaluated relative to sociodemographics (age, sex, race/ethnicity, marital status, region), socioeconomics (education, income, employment, insurance), health behaviors (diet, exercise, smoking, alcohol), and health/functioning (emotional support, BMI, mental/physical health). Overall, insufficient sleep was associated with being female, White or Black/African-American, unemployed, without health insurance, and not married; decreased age, income, education, physical activity; worse diet and overall health; and increased household size, alcohol, and smoking. These factors should be considered as risk factors for insufficient sleep.

## Introduction

Sleep is an important public health issue, with chronic sleep insufficiency linked to many societal problems including motor vehicle accidents, industrial disasters, and medical and other occupational errors. Poor sleep is associated with chronic diseases including hypertension, depression, diabetes, and obesity, as well as higher mortality rates (1–3). Increased awareness of the importance of healthy sleep is needed (1). Previous research in other domains of healthy behavior have shown that in order to meet these demands, a more complete understanding of social and behavioral “determinants” is needed (4, 5).

Social determinants of sleep have been previously explored; the very same social characteristics associated with good health such as a high level of education, being married, and being employed are also associated with higher sleep quality and healthy sleep duration (6, 7). Income plays a role in perceived sleep quality – as income decreases, sleep complaints increase (6, 8). Individuals with less education are more likely to experience insomnia (9) and report sleep disturbance (6). Living in an inner city environment has a negative effect on sleep duration – persons who live in unhealthy neighborhoods have an increased risk of unhealthy sleep (10–12). Blacks are at an increased risk for short sleep and/or poor sleep as compared to whites (13–15), and insufficient sleep among blacks is associated with greater cardiometabolic risk (7, 16). Employment also has an effect on sleep. The unemployed report poorer sleep than those who are employed (6). It is clear that socioeconomic variables are related to sleep complaints in general, such that increased socioeconomic status is associated with fewer sleep complaints.

Although the preponderance of studies assessing the societal implications of sleep loss have focused on short sleep duration, rather than perceived insufficient sleep, these likely represent overlapping, though separate, constructs (2, 17). Very few studies have specifically addressed health effects of unmet sleep need independent of sleep duration. Shankar et al. (18) and Altman and colleagues (19) found that insufficient sleep was associated with cardiometabolic health. Earlier studies also studied perceived sleep insufficiency, finding associations with and reported poor general health, functional deficits (2005), and increased cortisol secretion and abnormal growth hormone metabolism (20). For our purposes, insufficient sleep is defined as reduction in sleep time of a magnitude associated with a negative health outcome (2).

Although many associations have been documented, few studies have evaluated a broad array of social, behavioral, and health predictors in a nationally representative sample. Accordingly, the present study evaluated the social and behavioral determinants of insufficient sleep in a nationally representative sample. In this secondary data analysis, we utilized the 2009 behavioral risk factor surveillance system (BRFSS), which collected data on insufficient sleep from >300,000 individuals from all 50 states. Our hypotheses were that rates of insufficient sleep would be related to sociodemographic factors, socioeconomic factors, health behaviors, and health status. Further, we hypothesize that in fully adjusted analyses, effects will be attenuated but will remain significant, indicating that all of these factors contribute unique variance to insufficient sleep.

## Materials and Methods

Data from the 2009 BRFSS were used (21). The BRFSS is an annual, state based, random-digit-dialed telephone survey of adults in the United States. It is conducted by the Centers for Disease Control and Prevention and is designed to monitor health-related behaviors in the general population. For the present analyses, data from all 50 states and multiple territories were included. Response rate varied by state, with a median of 53.86% and a range of 37.90% (Oregon) to 72.78% (Guam).

All analyses are weighted to account for differences in selection probability, lack of coverage or response, number of residential telephone lines in the home of each participant, and number of adults in the household. This increases generalizability of results by accounting for factors of location and accessibility, and characteristics including age, sex, and ethnicity (22).

### Sleep

The outcome of interest was “perceived insufficient rest or sleep” (insufficient sleep). This was measured using the item, “During the past 30 days, for about how many days have you felt you did not get enough rest or sleep?” Responses were divided by 4.28 to convert to days/week in order to facilitate interpretation.

### Sociodemographics

Age was assessed as a continuous variable and categorized into groups. Sex was assessed categorically. Race/ethnicity was categorized as “Non-Hispanic White,” “Black/African-American,” “Hispanic/Latino,” “Asian/Other,” or “Multiracial.” Marital status was assessed as “Married,” “Never Married,” “Part of an Unmarried Couple,” “Divorced,” “Widowed,” or “Separated.” Household size was assessed as a continuous variable. Census regions were determined based on state.

### Socioeconomics

Household income level was assessed categorically. Education level was assessed as either “Less Than High School,” “High School Graduate,” “Some College,” or “College Graduate.” Employment status was assessed as “Employed,” “Retired,” “Homemaker,” “Student,” “Unemployed,” or “Unable to Work.” Access to health insurance was assessed binomially (yes/no).

### Health behaviors

Alcohol use was assessed categorically (heavy drinking: >1 drink/day in women, >2 drinks/day in men) and continuously (drinks/month). Smoking was assessed as “Never,” “Former,” “Current Some Days,” or “Current Every Day.” Diet was assessed as daily servings of fruits/vegetables. Physical activity was assessed several ways. Categorical assessments evaluated the presence of any exercise in the past 30 days, and also assessed physical activity at work. Physical activity was assessed continuously as computed hours/day of moderate/vigorous activity.

### Health and functioning

Availability of emotional support was categorized as “Never,” “Rarely,” “Sometimes,” “Usually,” or “Always.” Body mass index was categorized as Underweight (<18), Normal (18–25), Overweight (25–30), or Obese (>30). Although self-reported height and weight may be unreliable due to reporting bias, these data have demonstrated their utility and have been accepted in other studies (23–27). Overall health was assessed categorically (“Very Poor,” “Poor,” “Good,” “Very Good,” or “Excellent”) and continuously as number of days in the past 30 days of subjective poor physical and mental health.

### Statistical analyses

These variables were chosen *a priori*, as previous studies have found that many of these variables were related to sleep (6, 28). Complete case analysis was implemented. The general analytic strategy was to build a parsimonious model for a subsample of the data and then evaluate the model performance on the remaining sample. To facilitate this, the data were randomly split in half with one partition being treated as a training dataset (for model development) and the second partition being treated as a testing dataset (for evaluating model fit). Model development was conducted using stochastic gradient boosted modeling (GBM). GBM is a statistical (or machine) learning technique that combines the simplicity of decision trees with a high level of predictive accuracy, which is achieved through iteratively growing a large number of decision trees with each subsequent tree fit to the regularized (i.e., shrinkage) residuals from the previous tree. The number of trees, the shrinkage rate (learning rate), and number of terminal nodes in each tree (interaction depth) can be controlled and set empirically via cross-validation to maximize predictive accuracy. In these analyses, 10-fold cross-validation was used to determine the optimal [minimized root mean square error (RMSE)] interaction depth and number of decision trees. A shrinkage rate of 0.01 was used per. In the training dataset, 3000 gradient boosted trees were fit to the sleep insufficiency outcome using all 23 predictors. The primary measure from this analysis was relative influence. The relative influence of each predictor was calculated as the average across all trees of the empirical improvement (via RMSE) of the model from splitting a decision tree on a predictor. Model fit was assessed by examining the *R*^2^ (proportion of variance explained; *R*^2^) in the testing dataset from predictions using the coefficients from the training dataset. A non-parametric percentile bootstrap of 1000 replications was used to compare *R*^2^ between models. Stability of the selection of influential predictors was assessed using Pearson correlations of the relative influences between models.

The GBM analyses were conducted using the *gbm* package in R version 2.14.0. All other analyses were conducted using STATA/SE version 12 (STATA Corp, College Station, TX, USA).

## Results

### Sample characteristics

The sample included a total of *N* = 323,047 individuals, representing all 50 states, Washington, DC, Puerto Rico, US Virgin Islands, and Guam. Characteristics of the sample can be found in Table 1. Response to the insufficient sleep item was very high, with 1.8% not responding. Analysis of characteristics of non-responders showed no notable differences to responders (Table S1 in Supplementary Material). Insufficient sleep for at least one night/week was reported in over half the sample, with >10% reporting insufficient sleep seven nights/week.

**Table 1:** **Characteristics of the total sample**.

Variable	Category	Values
Insufficient sleep (days/week)	Mean (SD)	2.00 (2.33)
Age	18–24 (%)	9.76
	25–29 (%)	8.07
	30–34 (%)	11.21
	35–39 (%)	9.48
	40–44 (%)	11.26
	45–49 (%)	9.59
	50–54 (%)	10.64
	55–59 (%)	7.92
	60–64 (%)	7.07
	65–69 (%)	4.80
	70–74 (%)	3.65
	75–79 (%)	3.17
	80+ (%)	3.36
Sex	Female (%)	50.57
	Male (%)	49.43
Race/ethnicity	White (%)	69.34
	Black/African-American (%)	9.72
	Hispanic/Latino (%)	14.34
	Asian/other (%)	5.06
	Multiracial (%)	1.54
Household size	Mean (SD)	3.18 (1.60)
Education	Less than high school (%)	9.23
	High school (%)	26.48
	Some college (%)	27.13
	College graduate (%)	37.16
Income	<$10,000 (%)	4.84
	$10,000–$15,000 (%)	4.89
	$15,000–$20,000 (%)	6.81
	$20,000–$25,000 (%)	8.43
	$25,000–$35,000 (%)	10.38
	$35,000–$50,000 (%)	14.41
	$50,000–$75,000 (%)	16.59
	$75,000+ (%)	33.66
Marital status	Married (%)	62.90
	Divorced (%)	8.76
	Widowed (%)	5.23
	Separated (%)	1.96
	Never married (%)	17.35
	Part of an unmarried couple (%)	3.80
Census region	West (%)	23.40
	Midwest (%)	22.59
	South (%)	35.57
	Northeast (%)	17.23
	Other (%)	1.21
Employment status	Employed (%)	60.72
	Retired (%)	14.48
	Homemaker (%)	7.35
	Student (%)	4.36
	Unemployed (%)	8.20
	Unable to work (%)	4.90
Health insurance	Yes (%)	85.64
	No (%)	14.36
Fruits and vegetables	<1/day (%)	4.60
	1–3/day (%)	35.77
	3–5/day (%)	35.67
	5+/day (%)	23.97
Moderate exercise (h/day)	Mean (SD)	0.13 (0.17)
Vigorous exercise (h/day)	Mean (SD)	0.08 (0.13)
Any exercise	No (%)	23.12
	Yes (%)	76.88
Physical activity at work	Mostly sitting/standing (%)	39.84
	Mostly walking (%)	12.98
	Mostly heavy labor (%)	7.68
	Not employed (%)	39.50
Number of drinks	Mean (SD)	12.10 (36.40)
Heavy drinking	No (%)	94.61
	Yes (%)	5.39
Smoking	Never (%)	57.03
	Former (%)	25.14
	Some days (%)	5.23
	Every day (%)	12.61
Emotional support	Always (%)	48.07
	Usually (%)	31.06
	Sometimes (%)	12.97
	Rarely (%)	3.98
	Never (%)	3.91
BMI category	Underweight (%)	1.48
	Normal (%)	33.67
	Overweight (%)	36.66
	Obese (%)	28.19
Days poor physical health	Mean (SD)	3.52 (7.75)
Days poor mental health	Mean (SD)	3.48 (7.58)
Overall health	Excellent (%)	21.53
	Very good (%)	34.25
	Good (%)	29.31
	Poor (%)	11.01
	Very poor (%)	3.89

### Cross-validation

In the training dataset, 10-fold cross-validation was used to determine the appropriate number of decision trees and interaction depth of the models. An interaction depth of 7 was found to minimize the cross-validated RMSE from the models. A separate GBM was then conducted at an interaction depth of 7 with 3000 initial trees. From this model, tree number 1809 was found to minimize the RMSE. The initial model showed that the number of days with poor mental health was a highly influential variable in the analysis, as such additional models without this variable were also fit to determine model robustness to this variable’s absence. In the training dataset without this variable, 2013 trees were found to minimize the RMSE.

### Insufficient sleep and sociodemographics

**Figure 1:**
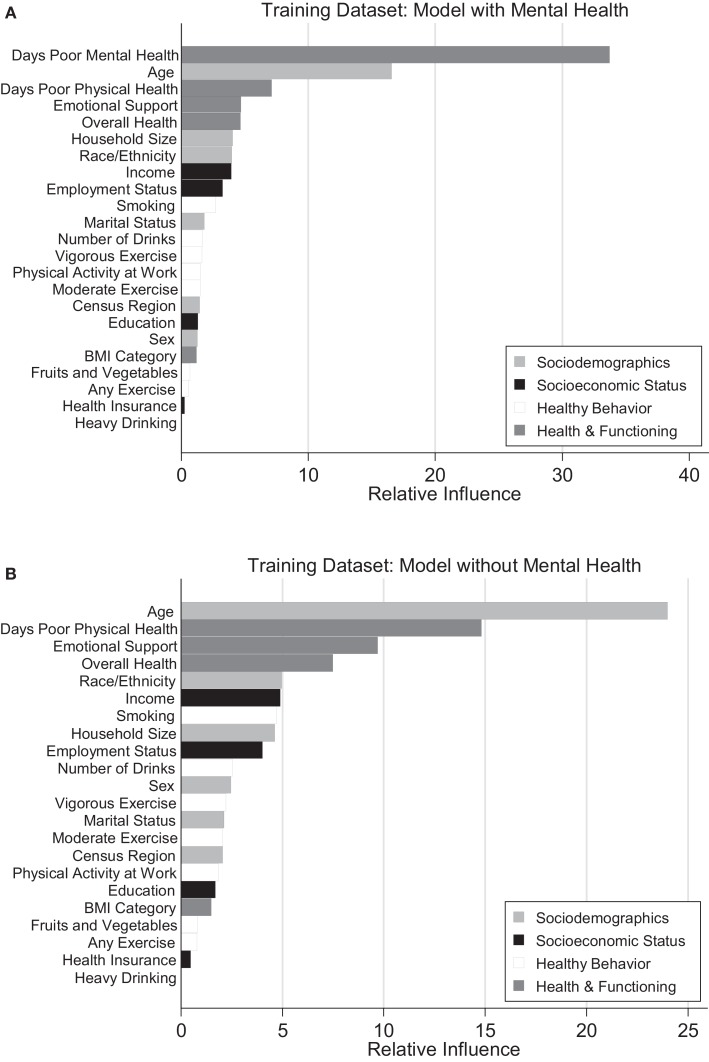
**Rrelative influence of predictors from GBM in models with and without mental health predictor**. **(A)** Model with mental health. **(B)** Model without mental health.

Figure 1 represents a graphical depiction of variable influence for a model with (Figure 1A) and without (Figure 1B) the mental health predictor.

#### Age

**Figure 2:**
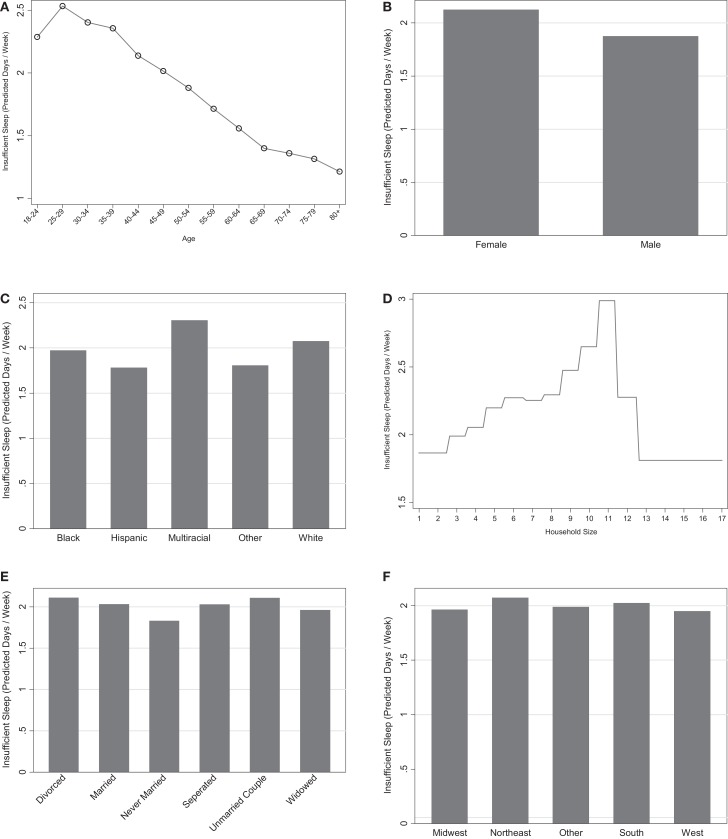
**Marginal predictions for sociodemographic variables**. **(A)** Age. **(B)** Sex. **(C)** Race/ethnicity. **(D)** Household size. **(E)** Marital status. **(F)** Census region.

Age was the most influential sociodemographic variable and second most influential variable overall (Figures 1A,B). There was a general decrease in insufficient sleep across age groups, with the highest levels reported in younger adults and the lowest levels seen in the oldest adults. These relationships are graphically represented in Figure 2A.

#### Sex

Overall, women were more likely to report insufficient sleep than men (Figure 2B). The influence of this variable was low to moderate in both models with and without the mental health predictor (1.3 and 2.5, respectively), suggesting that gender is one of the least influential variables for predicting sleep insufficiency.

#### Race/Ethnicity

Race was consistently in the top one-third of predictors in the models (Figures 1A,B). The Hispanic-Latino, Black/African-American, and Asian/Other groups were less likely to report insufficient sleep than the White group, and the multiracial groups were more likely to report insufficient sleep. These relationships are displayed graphically in Figure 2C.

#### Household Size

Household size was also in the top one-third of predictors in the models (Figures 1A,B). Overall, larger household size was associated with greater sleep insufficiency (Figure 2D).

#### Marital Status

Marital status had a low to moderate level of influence on sleep insufficiency in both models (1.8 and 2.1). Respondents who were never married reported the lowest levels of insufficient sleep, followed by those who were widowed. Married and separated respondents reported the next highest levels; and respondents living with a partner or divorced were the most likely to report insufficient sleep. This is displayed graphically in Figure 2E.

#### Census Region

Census region was not considered to be an influential variable in predicting insufficient sleep, such that its relative influence was consistently in the bottom one-third of predictors. Respondents from the South and the Northeast were more likely to report insufficient sleep compared to the other regions. This is represented graphically in Figure 2F.

#### Summary of Influence of Sociodemographics

Age, race/ethnicity, and household size were considered to be good predictors of insufficient sleep based on the models with and without mental health. As a whole, the sociodemographic variables had total relative influences of 29.2 and 40.3 making them the second most important variable set in predicting insufficient sleep in the model with mental health and the most important variable set in the model without mental health (Table 2).

**Table 2:** **Summary of relative influence between predictor groups within training dataset models**.

	Sociodemographics	Socioeconomic status	Healthy behaviors	Health and functioning
Model without mental health	29.2	8.9	10.4	51.6
Model with mental health	40.3	11.1	15.1	33.5

### Insufficient sleep and socioeconomic status

#### Income

**Figure 3:**
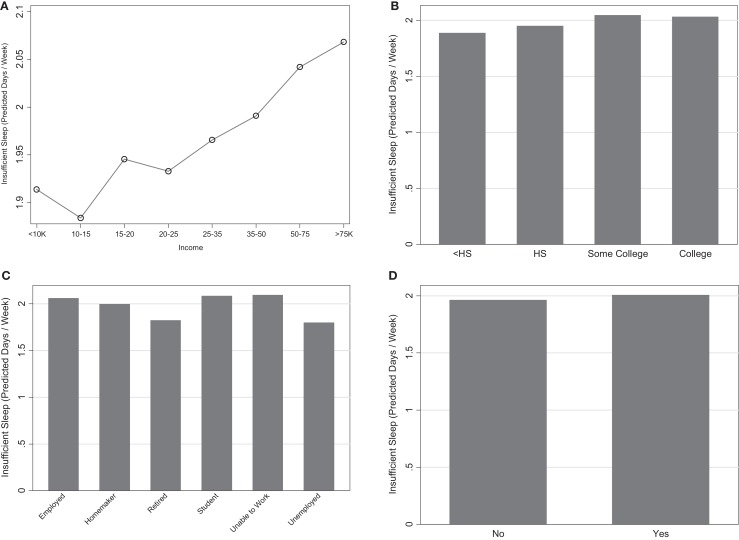
**Marginal predictions for socioeconomic status variables**. **(A)** Income. **(B)** Education. **(C)** Employment. **(D)** Health insurance.

Income was a highly influential variable in both models (top one-third) such that there was an overall increase in insufficient sleep associated with increased income (Figure 3A).

#### Education

Respondents with less than a high school education reported the lowest levels of insufficient sleep, followed by high school graduates (Figure 3B). Both of these reflected lower levels of insufficient sleep than college graduates. This variable, however, was one of the least influential and thus is not likely to be a strong predictor in explaining insufficient sleep.

#### Employment Status

Retired and unemployed individuals had the least amount of less insufficient sleep (Figure 3C). Students, the employed, and those unable to work had the highest levels of insufficient. Employment status was a moderately influential variable in both models (3.2 and 4.0). This is represented in Figures 1A,B.

#### Health Insurance

Lack of health insurance was associated with slightly less reports of insufficient sleep (Figure 3D); however, this variable was the second least influential variable in the model and is not considered to be a good predictor of insufficient sleep.

#### Summary of Influence of Socioeconomic Status

The most influential variables from this variable set were income and employment. As a whole, the socioeconomic status variables had the least influence of any variables in both models (summated relative influences of 8.9 and 11.1) (Table 2).

### Insufficient sleep and healthy behavior

#### Healthy Diet

**Figure 4:**
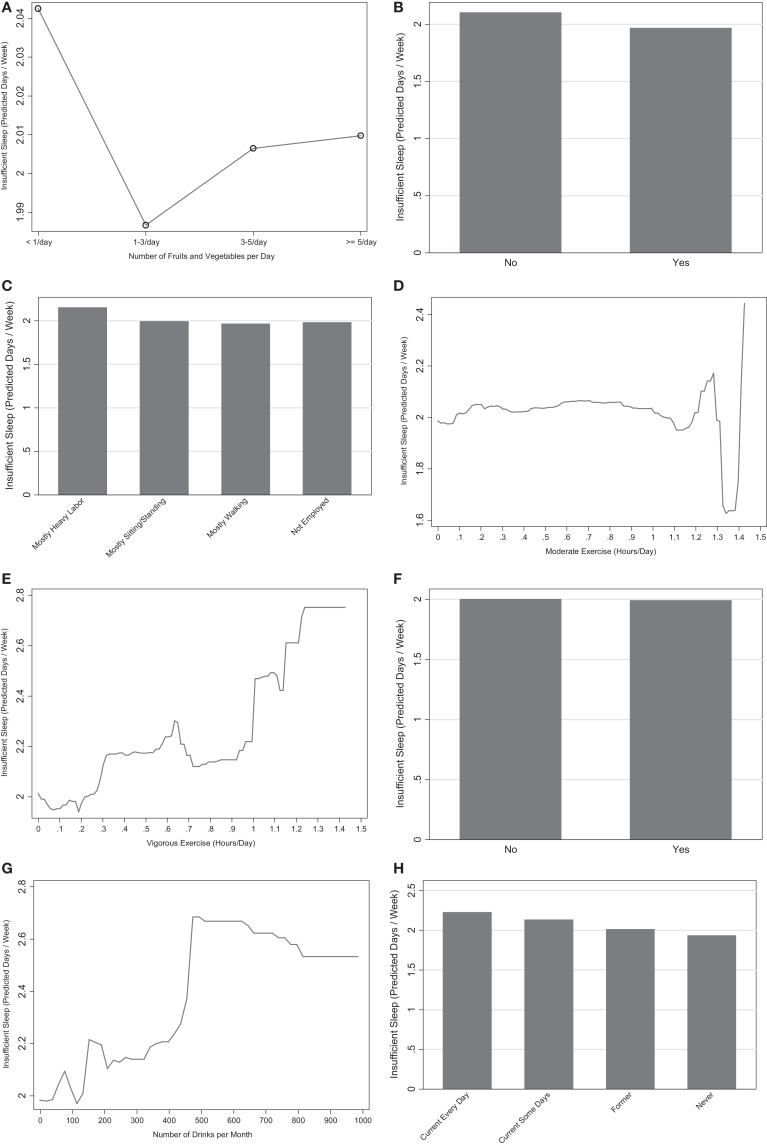
**Marginal predictions for healthy behavior variables**. **(A)** Fruits and vegetables. **(B)** Any exercise. **(C)** Physical activity at work. **(D)** Moderate exercise. **(E)** Vigorous exercise. **(F)** Heavy alcohol. **(G)** Number of drinks. **(H)** Smoking.

Consuming <1 serving of fruits/vegetables/day was associated with higher levels of insufficient sleep (Figure 4A), though the differences in insufficient sleep between the fruit/vegetable serving groups was trivial (<0.05 days/week). Additionally, this variable had very low relative influence.

#### Exercise

The presence of any exercise in the past month was associated with decreased insufficient sleep (Figure 4B). Respondents who were not employed or were employed in jobs that involve mostly walking were less likely to report insufficient sleep than respondents whose jobs were mostly sitting/standing, and those whose jobs were mostly hard labor reported more (Figure 4C). When exercise was assessed continuously, both moderate and vigorous exercise were positively associated with increased sleep insufficiency, though the marginal predictions for <1.2 h/day of moderate exercise showed differential results likely due to insufficient *N* at these extremes (Figures 4D,E). Overall, the continuous measures of exercise amount were moderately influential predictors in the models with and without the mental health variable. The discrete variables about any exercise and physical activity at work were not influential in the model without the mental health variable.

#### Alcohol

Heavy drinking was the least influential variable in all analyses and had negligible differences in insufficient sleep between alcohol groups (Figure 4F). When alcohol intake was assessed continuously, increased alcohol intake was associated with more insufficient sleep (Figure 4G) and was moderately influential.

#### Smoking

Being a current smoker (some days or every day) was associated with increased insufficient sleep, relative to those who were former smokers or never smoked (Figure 4H). Smoking had a moderate to high influence in both models and was the most influential variable pertaining to health behaviors.

#### Summary of Influence of Healthy Behavior

This variable set as a whole was the third most influential in both models (summated relative influences of 10.4 and 15.1) (Table 2). The variables pertaining to smoking, the number of drinks, and the continuous measures of exercise were all moderately influential predictors of insufficient sleep.

### Insufficient sleep, health, and functioning

#### Emotional Support

**Figure 5:**
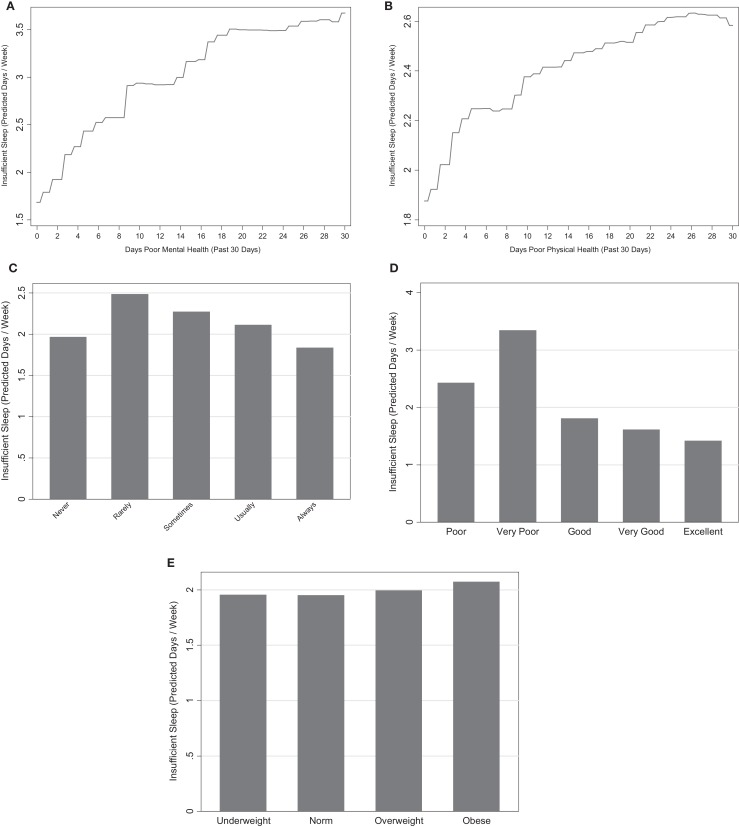
**Marginal predictions for health and functioning variables**. **(A)** Days poor mental health. **(B)** Days poor physical health. **(C)** Emotional support. **(D)** Overall health. **(E)** BMI category.

Emotional support was the fourth and third most influential variable in the model with and without mental health, respectively. All categories of emotional support were associated with increased insufficient sleep, relative to respondents who have support “Always.” Respondents who have support “Never” also had low levels of insufficient sleep relative to the remaining categories (Figure 5A).

#### Body Mass Index

Overweight and obese respondents had the highest levels of sleep insufficiency, though differences between BMI categories were slight (Figure 5B). BMI category was the fifth least influential variable in both models (1.2 and 1.5), indicating it as a poor predictor of insufficiency.

#### Overall Health

Worse overall health was associated with higher insufficient sleep (Figure 5C). Similarly, respondents who reported to be in the best overall health had the lowest amounts of insufficient sleep. The highest levels of insufficient sleep were associated with very poor health. Overall health was the fifth and fourth most influential variable in the models with and without the mental health variable, respectively.

#### Days of Poor Physical and Mental Health

Days of poor physical and mental health was strongly associated with insufficient sleep. Days of poor mental health was the most influential predictor (relative influence 33.8) and days of poor physical health was the third and second most influential variable in the two models (relative influences 7.1 and 14.8) (Figures 1A,B). Both variables exhibited a positive association with insufficient sleep such that those with more days of poor mental and physical health had higher levels of insufficient sleep (Figures 5D,E).

#### Summary of Influence of Health and Functioning

This variable set was the most influential in the model with mental health (summated relative influence 51.6) and the second most influential in the model without mental health (summated relative influence 33.5). All of the variables in this variable set with the exception of BMI were highly influential (Figures 1A,B).

### Stability of influential predictors

Additional analyses using gradient boosting in the testing dataset were used to examine the stability of the predictor selections from the training dataset. These relative influence values from the testing dataset were found to be highly correlated to the ones from the training dataset at 0.993 and 0.995 (Table 3). Within each dataset, the relative influences between the model with and without the mental health predictor were correlated at 0.976 in the training dataset and 0.982 in the testing dataset.

**Table 3:** **Correlation of relative influences between testing and training datasets**.

		Training		Testing	
		Model with mental health	Model without mental health	Model with mental health	Model without mental health
Training	Model with mental health	1.0			
	Model without mental health	0.976	1.0		
Testing	Model with mental health	0.993	–	1.0	
	Model without mental health	–	0.995	0.982	1.0

### Model fit

Model fit was assessed by using the coefficients discovered in the training dataset to predict values in the testing dataset. A percentile bootstrap was used to compare *R*^2^ between models. In both the testing and training datasets, the model *R*^2^ was significantly (*p* < 0.05) reduced in the model without mental health from 20.9 to 18.0% in the training dataset and from 20.4 to 17.3% in the testing dataset (Table 4). Among the models with the mental health predictor, the *R*^2^ in the training dataset did not significantly differ from the testing dataset; however, in the model without mental health the *R*^2^ was significantly (*p* < 0.05) reduced by 0.7% in the testing data (Table 4).

**Table 4:** **Comparisons of model *R*^2^ using a percentile bootstrap**.

	Training *R*^2^ (95% CI)	Testing *R*^2^ (95% CI)	Δ *R*^2^ (95% CI)
Model with mental health	0.209 (0.206, 0.213)	0.204 (0.200, 0.209)	0.005 (−0.001, 0.011)
Model without mental health	0.180 (0.176, 0.184)	0.173 (0.169, 0.177)	0.007 (0.001, 0.012)
Δ *R*^2^ (95% CI)	0.029 (0.024, 0.035)	0.031 (0.025, 0.037)	

## Discussion

The present study evaluated the predictors of insufficient sleep in a large epidemiologic sample. Nearly, all variables were significantly related to insufficient sleep to some degree, though some factors (e.g., overall mental health) were more strongly related than others (e.g., alcohol use). Taken together, these findings demonstrate that a myriad of social and behavioral factors contribute to insufficient sleep.

The proportion of variance explained by this relatively wide array of variables is restricted to approximately 17–20%. This is a relatively low figure, given the large number of potential factors included. It is possible that other factors not included, such as work schedule, number of jobs, beliefs and attitudes about sleep, and history of sleep, medical, and psychiatric disorders, may also play unique roles. Further, there is likely a high degree of measurement error in these survey items, contributing to a diminished predictive ability.

### Insufficient sleep and sociodemographics

We found a significant age effect; the level of insufficient sleep gradually decreased with age, with the highest levels in younger adults and the lowest levels in the oldest adults, replicating our previous finding (29). It is plausible that persons in the oldest age group may report the highest sleep quality because those with poor sleep quality associated with chronic illnesses may have died earlier or were unable to complete the survey.

Insufficient sleep was more common among women. This finding is in line with the majority of other studies (29–34), reporting that women have a higher need for sleep and more sleep disturbances than men. In our study, increasing household size was also associated with increased level of insufficient sleep. Even though we did not look at interaction between gender and household size, we speculate that increasing household size probably impacts women’s sleep more, as women are the primary caregivers in most families.

Our results show that, after adjusting for all covariates, the Hispanic/Latino, Black/African-American, and Asian/Other groups were less likely to report insufficient sleep than the White group, but multiracial individuals were more likely to report insufficient sleep. Results from the 2006 BRFSS similarly showed that Hispanic/Latino and Black/African-American individuals reported fewer complaints and multiracial individuals reported more complaints (6) and Hispanics had fewer days of insufficient sleep than African-Americans or Whites (33). Whites may be more willing to report symptoms, which have been shown in other large epidemiologic studies (34). Also, although we adjusted for many covariates, there could be other effects or interactions that are not modeled.

Divorced individuals reported increased insufficient sleep, relative to married individuals and those that were never married reported less insufficient sleep. These results are in agreement with previous studies reporting that divorced, separated, and widowed individuals are more likely to be short-sleepers compared to married individuals (30, 32, 35–37). Studies assessing the effect of poor sleep on marital satisfaction found that sleep problems reported by one or both spouses were associated with higher levels of marital unhappiness (38, 39). Thus, marriage may, overall, protect against insufficient sleep, but the reciprocal pathway is also plausible, i.e., sleep disruption/insufficiency may decrease marital harmony.

In adjusted analyses, responders from the South and the Northeast were more likely to report insufficient sleep. This finding is consistent with our previous study (40), which showed that those in the South are often most likely to report sleep disturbance and daytime fatigue, and those in the West are often least likely. It is plausible that environmental factors including noise, green space, and air pollution, which have been shown to contribute to obesity (41, 42), diabetes risk (43), and other chronic diseases (44), may contribute to sleep disturbance. However, studies are needed to examine these determinants in relation to insufficient sleep.

### Insufficient sleep and socioeconomic factors

We found important differences in socioeconomic factors, relative to insufficient sleep. These findings are in contrast to earlier studies (8, 14, 33, 35, 37, 45, 46). After adjustment for many covariates, the direction of the relationship was opposite of what has been seen in the past. For example, individuals in low-income groups ranging from <$10,000 to $50,000, with a high school education or less, retired, homemakers, and unemployed, and without health insurance reported less insufficient sleep.

There are a number of reasons why lower income was associated with less insufficient sleep. First, prior population-based studies are often limited by a sample that is not nationally representative. Second, we included a very wide array of possible confounding factors, which may account for many of the benefits of increased income (such as better health, employment, and health care); once the variance explained by these factors is removed, effects independent of known potential benefits of high socioeconomic status can be elucidated.

### Insufficient sleep and healthy behavior

Previous studies demonstrated that unhealthy behaviors such as smoking, physical inactivity, and heavy drinking are adversely associated with insufficient sleep (30, 36, 37, 46). Similarly, our results regarding alcohol consumption indicated that both heavy drinking as a categorical variable and increased amount of alcohol consumption as a continuous variable are associated with more sleep insufficiency. However, it is surprising that when we controlled for other covariates, heavy drinking was associated with less insufficient sleep. Our findings showing that current and former smokers are more likely to report insufficient sleep than non-smokers are in line with the results from previous studies (8, 33), since nicotine is a stimulant. Regarding healthy eating, consuming <1 or 1–3 servings of fruits/vegetables/day was not associated with insufficient sleep after adjustment for confounders. Although habitual diet may be related to sleep (47–50), there may not be any associations with broad measures of healthy diet.

### Insufficient sleep and healthy functioning

The findings of the present study are consistent with the literature that demonstrates that short sleep duration is closely related to overweight and obesity (2, 51–68). Although there are some theories to explain these associations such as calorie intake and fatigue (64), future studies should explore the relationship between sleep duration and BMI longitudinally to establish causation and to elucidate the underlying mechanisms. It should be noted that these results are consistent with a previous study that examined a subset of this same cohort and found that insufficient sleep was associated with obesity (19). In the study by Altman et al., the effect of insufficient sleep was accounted for by sleep duration, suggesting that it is short sleep, and not perceived sleep insufficiency *per se* that is related to obesity.

Regarding associations with poor mental and physical health, the positive association with days of poor mental functioning suggests that sleep may be an important indicator of psychiatric disorders. Indeed, much of the literature in this domain focuses on specific sleep disorders and mental health risks including obstructive sleep apnea (69), insomnia (70), and symptoms of daytime sleepiness (71–73). However, habitual sleep, without the presence of a sleep disorder, seems to play an important role. Previous studies have demonstrated the role of sleep duration and psychological health risk, but most have focused on specific populations including the military (74, 75). Less is known, however, about the role of physical health functioning and sleep duration.

There is limited data on self-rated overall health and sleep duration. One large-scale study found that individuals with longer or shorter sleep duration were more likely to report fair/poor health even after controlling for important factors including, age, education, and BMI (76). Similarly, data from the 2008 BRFSS revealed that insufficient sleep was positively associated with poor health status (77). Together, these findings suggest that insufficient sleep is an important contributor to overall health status.

Regarding emotional support, women who reported low emotional support were more likely to report poor sleep compared to those who scored high on emotional support (78). These findings were not significant for men, suggesting a gender interaction between emotional support and poor sleep. The present study found that low emotional support was associated with insufficient sleep, echoing these previous findings.

### Limitations

Several issues about our study warrant discussion. First, the concept of insufficient sleep is not clearly defined. Its definition depends on the individuals’ ability to correctly judge their own sleep need. Therefore, the interpretability of this item is limited. Further, the insufficient sleep question does not differentiate between “rest” and “sleep.” Second, sleep disorders such as obstructive sleep apnea and insomnia are prevalent and often undiagnosed. Our data provide no information on sleep disordered breathing, other sleep symptoms, medication usage, mental/physical conditions, shift work, or caffeine consumption. Third is the issue of non-response. BRFSS has a relatively low-response rate. Because it is impossible to determine whether responders and non-responders differed in any meaningful way, potential bias is mitigated through the use of a weighting scheme in the context of a sample size large enough so that all groups are well represented. Although weighting procedures accounted for non-response to the BRFSS in general relative to state, age, sex, etc., weighting procedures did not account for non-response to the sleep item. However, these effects are probably small in magnitude because, on average, <2% of the respondents did not respond to the sleep item, and non-response did not reach 5% in any specific sociodemographic category for any of these items. The issue of cultural breakdown and relative perception has not been addressed. Categories such as Hispanic/Latino and Asian/other do not indicate country of origin, which could play a role in sleep disturbances (79). Although weighting procedures accounted for non-response to the BRFSS in general relative to age, race, sex, etc., weighting procedures did not account for non-response to the sleep insufficiency item. However, assessment of responses to other variables based on non-response to sleep items did not yield any patterns of non-response. Fourth, the cross-sectional design of this study limits our ability to comment on causality. Finally, BRFSS data are collected from civilian, non-institutionalized population through telephone surveys. Therefore, the results of this study may not be generalizable to individuals living in households without landline telephones and institutionalized individuals such as military. Therefore, the results should be interpreted cautiously, bearing these limitations in mind. Nevertheless, this study shows that over half the sample reported having insufficient sleep for at least one night/week and over 10% of the sample reported persistent insufficient sleep for seven nights/week. Further investigation of this public health problem is warranted.

## Conclusion

A wide array of social and behavioral factors play a significant role in reports of insufficient sleep, as it is experienced in a large, diverse, nationally representative sample. Overall, the variable most closely related to insufficient sleep was general mental health, though other variables, such as general physical health, age, race/ethnicity, emotional support, and household size, were also robustly associated with insufficient sleep. Overall, the social and behavioral determinants examined in this study accounted for approximately 17–20% of the variance of insufficient sleep, leaving room for future studies aimed at further clarifying which factors are most strongly related to insufficient sleep in the general population. These data may be useful for developing and targeting interventions to alleviate the increased risk of morbidity and mortality.

## Conflict of Interest Statement

The authors declare that the research was conducted in the absence of any commercial or financial relationships that could be construed as a potential conflict of interest.

## Supplementary Material

The Supplementary Material for this article can be found online at http://journal.frontiersin.org/article/10.3389/fneur.2015.00112/abstract
