# Supplementary material for: Social and Behavioral Determinants of Perceived Insufficient Sleep
Source: Front Neurol. 2015 Jun 5;6:112. doi: 10.3389/fneur.2015.00112 (PMC4456880; doi:10.3389/fneur.2015.00112)
Supplement: Supplementary file 1 [file table_1.docx]

**Supplementary Table 1.** Comparison of individuals missing data to those retained in analyses.

| Variable | Category | Values |  |
| --- | --- | --- | --- |
|  |  | Non-Missing  N=323,047 | Missing N=109,560 |
| Age | 18-24 (%) | 9.76% | 16.87% |
|  | 25-29 (%) | 8.07% | 6.80% |
|  | 30-34 (%) | 11.21% | 7.95% |
|  | 35-39 (%) | 9.48% | 7.17% |
|  | 40-44 (%) | 11.26% | 8.49% |
|  | 45-49 (%) | 9.59% | 7.21% |
|  | 50-54 (%) | 10.64% | 8.25% |
|  | 55-59 (%) | 7.92% | 6.58% |
|  | 60-64 (%) | 7.07% | 6.74% |
|  | 65-69 (%) | 4.80% | 5.78% |
|  | 70-74 (%) | 3.65% | 5.29% |
|  | 75-79 (%) | 3.17% | 5.40% |
|  | 80+ (%) | 3.36% | 7.47% |
| Sex | Female (%) | 50.57% | 53.65% |
|  | Male (%) | 49.43% | 46.35% |
| Race/Ethnicity | White (%) | 69.34% | 62.93% |
|  | Black/African-American (%) | 9.72% | 11.90% |
|  | Hispanic/Latino (%) | 14.34% | 17.39% |
|  | Asian/Other (%) | 5.06% | 6.11% |
|  | Multiracial (%) | 1.54% | 1.68% |
| Household Size | Mean (SD) | 3.18 ± 1.59 | 3.11 ± 1.72 |
| Education | Less Than High School (%) | 9.23% | 15.64% |
|  | High School (%) | 26.48% | 33.42% |
|  | Some College (%) | 27.13% | 24.56% |
|  | College Graduate (%) | 37.16% | 26.38% |
| Income | <$10,000 (%) | 4.84% | 8.90% |
|  | $10,000-$15,000 (%) | 4.89% | 7.46% |
|  | $15,000-$20,000 (%) | 6.81% | 9.81% |
|  | $20,000-$25,000 (%) | 8.43% | 11.09% |
|  | $25,000-$35,000 (%) | 10.38% | 12.15% |
|  | $35,000-$50,000 (%) | 14.41% | 14.04% |
|  | $50,000-$75,000 (%) | 16.59% | 12.99% |
|  | $75,000+ (%) | 33.66% | 23.56% |
| Marital Status | Married (%) | 62.90% | 53.43% |
|  | Divorced (%) | 8.76% | 8.16% |
|  | Widowed (%) | 5.23% | 9.23% |
|  | Separated (%) | 1.96% | 2.07% |
|  | Never Married (%) | 17.35% | 23.71% |
|  | Part of an Unmarried Couple (%) | 3.80% | 3.40% |
| Census Region | West (%) | 23.40% | 21.35% |
|  | Midwest (%) | 22.59% | 18.34% |
|  | South (%) | 35.57% | 38.29% |
|  | Northeast (%) | 17.23% | 20.49% |
|  | Other (%) | 1.21% | 1.53% |
| Employment Status | Employed (%) | 60.72% | 46.80% |
|  | Retired (%) | 14.48% | 20.80% |
|  | Homemaker (%) | 7.35% | 9.06% |
|  | Student (%) | 4.36% | 6.62% |
|  | Unemployed (%) | 8.20% | 9.99% |
|  | Unable to Work (%) | 4.90% | 6.73% |
| Health Insurance | Yes (%) | 85.64% | 81.64% |
|  | No (%) | 14.36% | 18.36% |
| Fruits and Vegetables | <1 Per Day (%) | 23.97% | 22.79% |
|  | 1-3 Per Day (%) | 4.60% | 7.82% |
|  | 3-5 Per Day (%) | 35.77% | 36.12% |
|  | 5+ Per Day (%) | 35.67% | 33.27% |
| Moderate Exercise (hrs/day) | Mean (SD) | 0.128 ± 0.167 | 0.126 ± 0.188 |
| Vigorous Exercise (hrs/day) | Mean (SD) | 0.079 ± 0.132 | 0.071 ± 0.148 |
| Any Exercise | No (%) | 23.12% | 29.51% |
|  | Yes (%) | 76.88% | 70.49% |
| Physical Activity at Work | Mostly Sitting / Standing (%) | 39.84% | 24.01% |
|  | Mostly Walking (%) | 12.98% | 8.98% |
|  | Mostly Heavy Labor (%) | 7.68% | 5.51% |
|  | Not Employed (%) | 39.50% | 61.51% |
| Number of Drinks | Mean (SD) | 12.1 ± 36.1 | 9.1 ± 33.3 |
| Heavy Drinking | No (%) | 94.61% | 95.91% |
|  | Yes (%) | 5.39% | 4.09% |
| Smoking | Never (%) | 57.03% | 58.00% |
|  | Former (%) | 12.61% | 13.04% |
|  | Some Days (%) | 5.23% | 5.29% |
|  | Every Day (%) | 25.14% | 23.67% |
| Emotional Support | Always (%) | 48.07% | 48.30% |
|  | Usually (%) | 31.06% | 26.21% |
|  | Sometimes (%) | 12.97% | 14.57% |
|  | Rarely (%) | 3.98% | 4.78% |
|  | Never (%) | 3.91% | 6.14% |
| BMI Category | Underweight (%) | 33.67% | 37.67% |
|  | Normal (%) | 1.48% | 2.39% |
|  | Overweight (%) | 36.66% | 35.06% |
|  | Obese (%) | 28.19% | 24.89% |
| Days Poor Physical Health | Mean (SD) | 3.52 ± 7.74 | 3.93 ± 8.43 |
| Days Poor Mental Health | Mean (SD) | 3.48 ± 7.57 | 3.55 ± 7.97 |
| Overall Health | Excellent (%) | 21.53% | 18.33% |
|  | Very Good (%) | 34.25% | 28.84% |
|  | Good (%) | 29.31% | 32.95% |
|  | Poor (%) | 11.01% | 14.43% |
|  | Very Poor (%) | 3.89% | 5.44% |
